# Supplementary figures and images for: VirION2: a short- and long-read sequencing and informatics workflow to study the genomic diversity of viruses in nature
Source: PeerJ. 2021 Mar 30;9:e11088. doi: 10.7717/peerj.11088 (PMC8018248; doi:10.7717/peerj.11088)

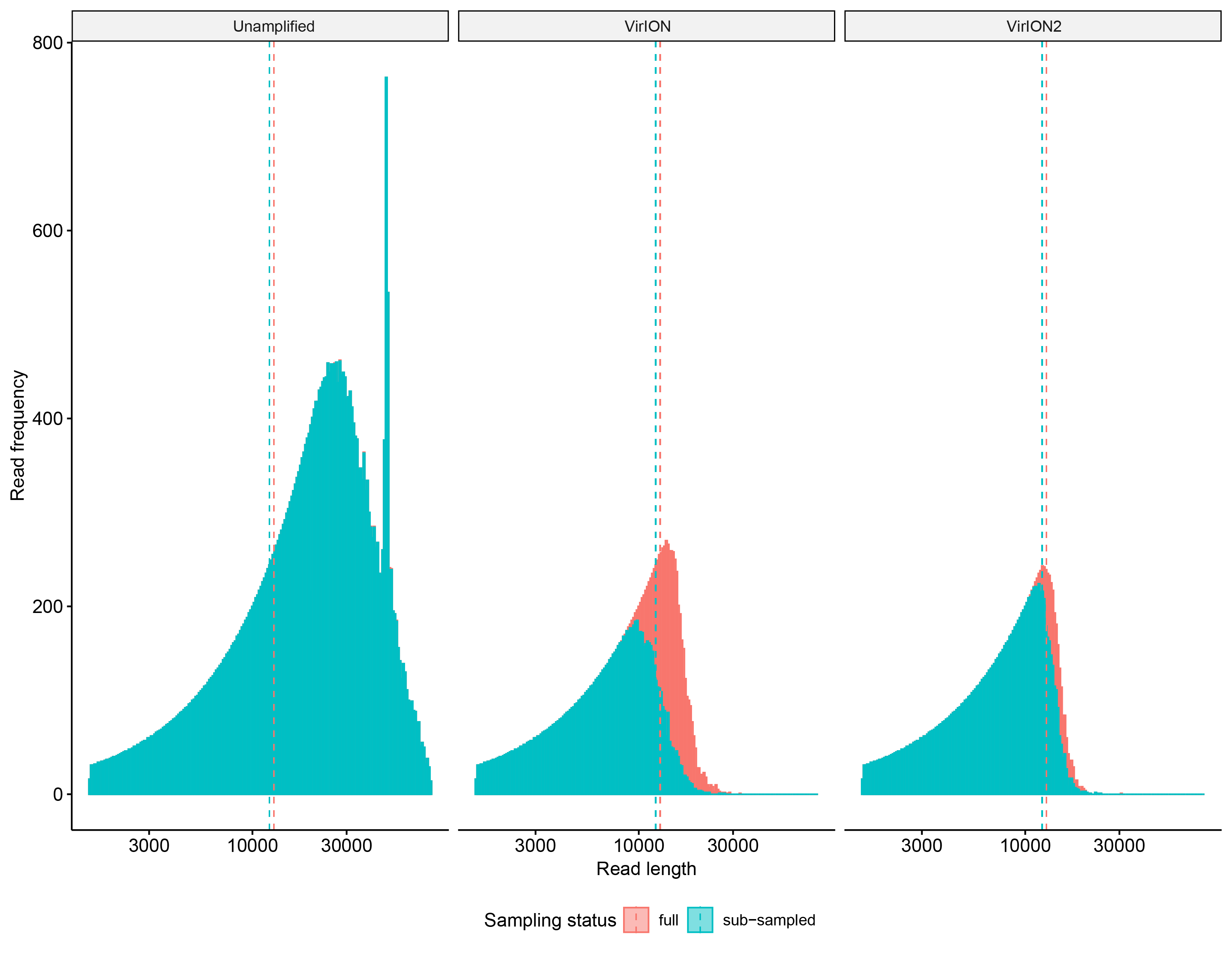

Supplement: Supplemental Information 1 — Frequency histograms depicting the read size distribution between the three long-read libraries: ‘raw’ (i.e., unamplified input DNA), VirION, and VirION2. Each library type is represented in its own facet, with both full (pastel red) and sub-sampled (turquoise) size distributions overlapping. Dotted lines and their associated colors denote the median value of each distribution. [file peerj-09-11088-s001.png]

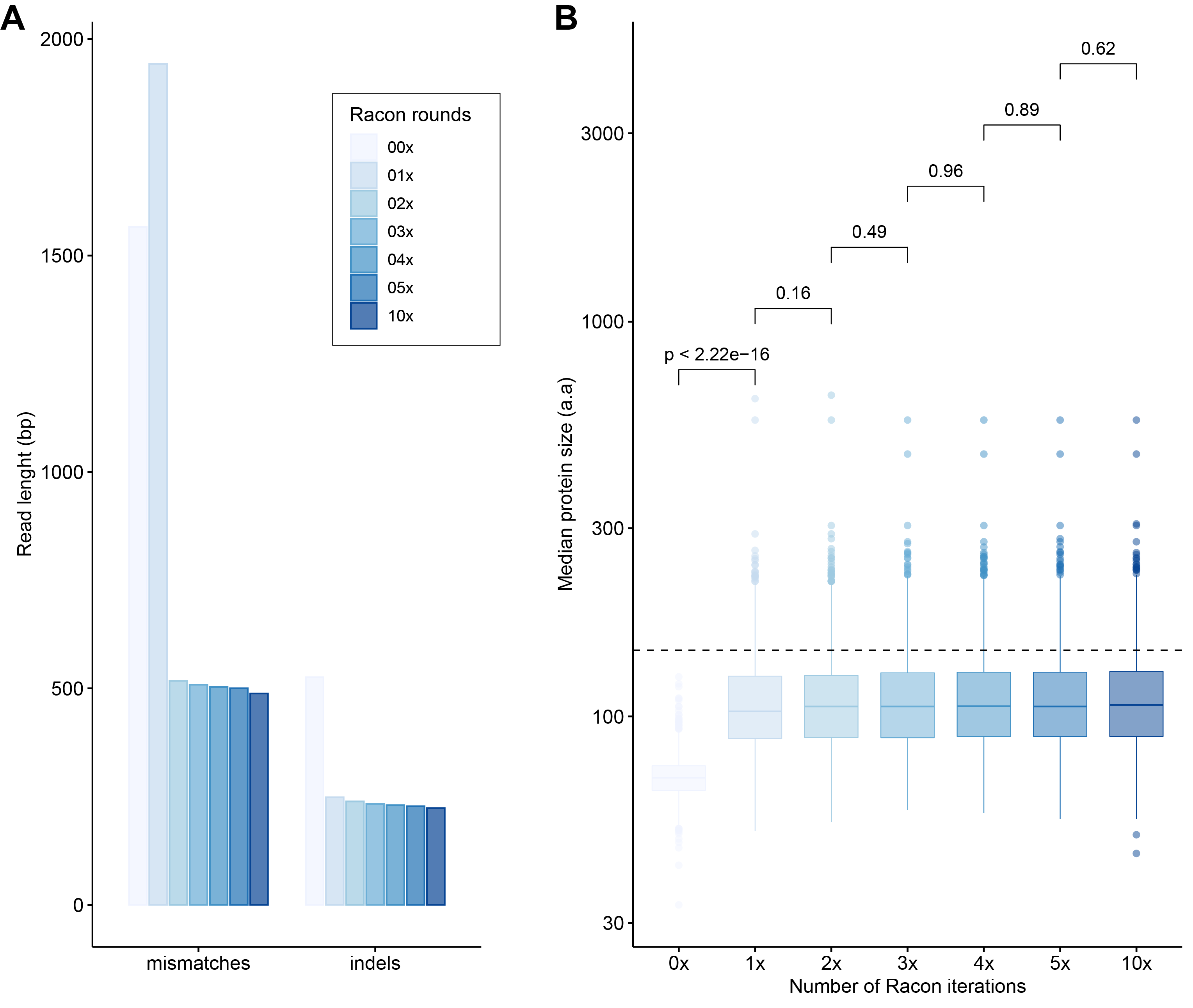

Supplement: Supplemental Information 2 — (A) Bar chart depicting the number of mismatches and insertion/deletions (indels), grouped by number of Racon rounds (on the x-axis) across increasing iterations of Racon polishing of long-read assemblies. From light to dark blue, increasing color saturation corresponds to the increasing number of Racon polishing rounds (also applicable to panel B). (B) Boxplot depicting the impact of consecutive rounds of Racon (x-axis) on predicted protein sizes (y-axis, measured in amino acids denoted as ‘aa’). The horizontal dotted line represents the median protein size of the corresponding short-read assembly (142 amino acids). [file peerj-09-11088-s002.png]

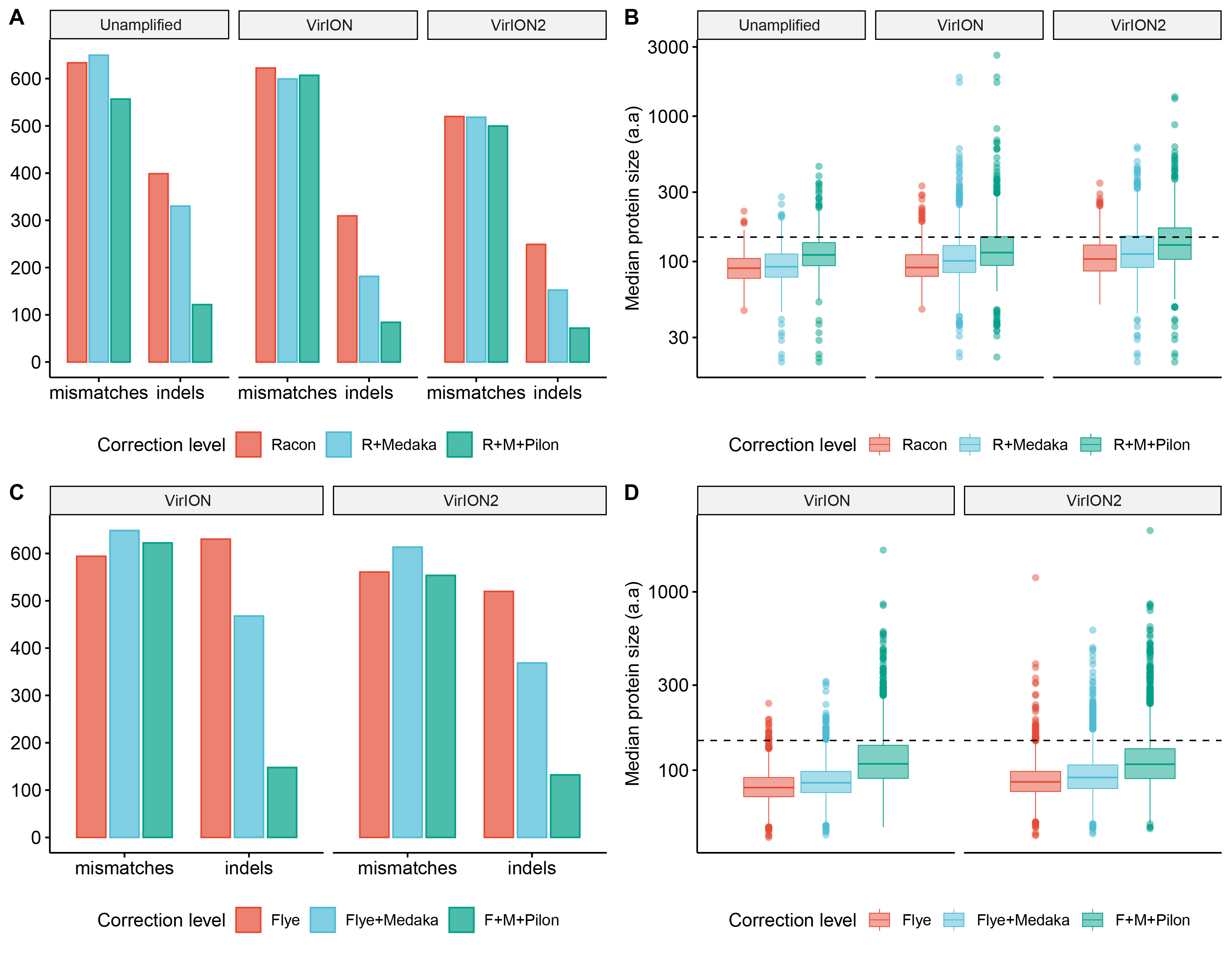

Supplement: Supplemental Information 3 — (A) Bar chart depicting the number of mismatches and insertion/deletions (indels) from the OLC strategy, grouped according to increasing correction (on the x-axis), separated by library method (raw, VirION, VirION2). (B) Boxplots depicting protein size distribution at each level of correction in the OLC strategy. The horizontal dotted line represents the median protein size of the corresponding short-read assembly (142 amino acids). (C) Bar chart depicting the number of mismatches and insertion/deletions (indels) from the ‘Flye’ strategy, grouped according to increasing correction (on the x-axis), separated by library method (VirION and VirION2). (D) Boxplots depicting protein size distribution at each level of correction in the ‘Flye’ strategy. The horizontal dotted line represents the median protein size of the corresponding short-read assembly (142 amino acids). [file peerj-09-11088-s003.png]

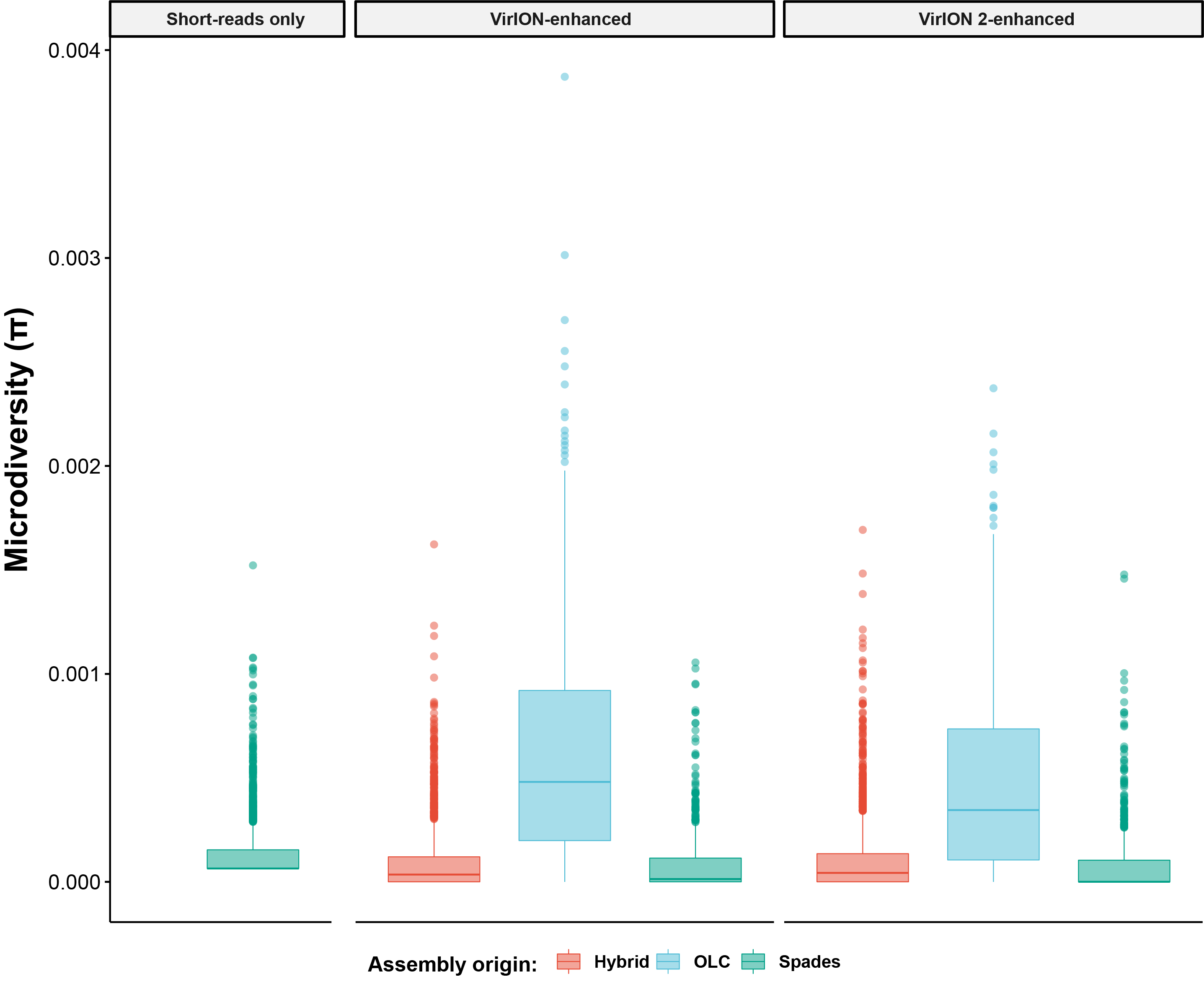

Supplement: Supplemental Information 4 — Boxplots displaying the per-genome microdiversity distribution across each virome assembly type (i.e.”,Short-reads only”, ”VirION-enhanced”, ”VirION2-enhanced”). Except for the ”Short-read only assembly”, data is further divided according to the origin of each constituent assembly (i.e., “Hybrid”, “OLC”, “Spades”). [file peerj-09-11088-s004.png]
