# Supplementary material for: VirION2: a short- and long-read sequencing and informatics workflow to study the genomic diversity of viruses in nature
Source: PeerJ. 2021 Mar 30;9:e11088. doi: 10.7717/peerj.11088 (PMC8018248; doi:10.7717/peerj.11088)
Supplement: Supplemental Information 6 [file peerj-09-11088-s006.docx]

**Supplementary Table 1:** Virus mock community composition and characteristics

| Phage | Taxonomy | GC (%) | Genome Size (kb) |
| --- | --- | --- | --- |
| *Pseudoalteromonas* phage HM1 | *Myoviridae* | 35.7 | 129.4 |
| *Pseudoalteromonas* phage HP1 | *Podoviridae* | 44.7 | 45.0 |
| *Pseudoalteromonas* phage HS2 | *Siphoviridae* | 40.2 | 38.2 |
